# The genome sequence of the Lunar Hornet, *Sesia bembeciformis* (Hübner 1806)

**DOI:** 10.12688/wellcomeopenres.19111.1

**Published:** 2023-03-01

**Authors:** Douglas Boyes, University of Oxford and Wytham Woods Genome Acquisition Lab, Darwin Tree of Life Barcoding collective, Wellcome Sanger Institute Tree of Life programme, Wellcome Sanger Institute Scientific Operations: DNA Pipelines collective, Tree of Life Core Informatics collective, William B.V. Langdon, Darwin Tree of Life Consortium

**Affiliations:** 1 UK Centre for Ecology and Hydrology, Wallingford, Oxfordshire, UK; 2 Department of Biology, University of Oxford, Oxford, Oxfordshire, UK

**Keywords:** Sesia bembeciformis, Lunar Hornet, genome sequence, chromosomal, Lepidoptera

## Abstract

We present a genome assembly from an individual male *Sesia bembeciformis* (the Lunar Hornet; Arthropoda; Insecta; Lepidoptera; Sesiidae). The genome sequence is 477.1 megabases in span. Most of the assembly is scaffolded into 31 chromosomal pseudomolecules, including the Z sex chromosome. The mitochondrial genome has also been assembled and is 16.1 kilobases in length. Gene annotation of this assembly on Ensembl has identified 15,843 protein coding genes.

## Species taxonomy

Eukaryota; Metazoa; Ecdysozoa; Arthropoda; Hexapoda; Insecta; Pterygota; Neoptera; Endopterygota; Lepidoptera; Glossata; Ditrysia; Sesioidea; Sesiidae; Sesiinae; Sesiini; *Sesia*; *Sesia bembeciformis* (Hübner 1806) (NCBI:txid287197).

## Background

The Lunar Hornet, S *esia bembeciformis* (Hübner 1806), is a moth of the family Sesiidae, commonly known as clearwings. This group are largely diurnal and named for their mostly scale-less, elongated wings which together with the banded, elongated abdomens of many species, contribute to their Batesian mimicry of aposematic Hymenoptera. *S. bembeciformis* is a relatively large species, with a wingspan of 31 to 48 mm ( Lastuvka & Lastuvka, 2001) and a broad abdomen with alternating yellow and black bands that enable it to mimic hornets ( *Vespa* spp).

S *esia bembeciformis* feeds mainly on willows ( *Salix* spp.) particularly *S. caprea* and *S. cinerea* ( Goossens, 2020; Henwood *et al.*, 2020; Lastuvka & Lastuvka, 2001). Some authors also state that the species is occasionally found on *Populus* spp. ( Henwood *et al.*, 2020; St John, 1890), which are the main foodplants of the related *S. apiformis* (which is also occasionally found on *Salix* spp.) ( Lastuvka & Lastuvka, 2001). *S. bembeciformis* can be encountered in a variety of habitats where these foodplants occur, and may prefer more open areas with scattered trees, in sunny positions ( Goossens, 2020). In the past it has occasionally been considered a pest of commercial willow crops – ( Johnson, 1874) wrote of it ‘doing considerable injury to the osier beds’.

In common with other clearwing species, *S. bembeciformis* larvae are rather nondescript, with creamy-white bodies, brown heads and prothoraic plates, and internal development on the foodplants ( Henwood *et al.*, 2020). Eggs are laid in small batches on the bark near the base of the tree, and larvae enter the trunk, often through a wound, where they feed initially on the sap ( Goossens, 2020). They can take several years to develop, hibernating over winter and moving deeper into the trunk, often down into the roots. Before their final hibernation, larvae prepare an exit hole in the trunk (leaving a thin layer of bark to cover it) before spinning a loose cocoon above it in spring and emerging in mid-summer. Adults are on the wing in June and July ( Randle *et al.*, 2019) and usually emerge early in the morning where they can be found resting on trunks above their emergence hole before engaging in active flight by day.

Males can be attracted to synthetic lures which mimic the sex pheromones produced by females, mostly during the morning before midday ( Pühringer, 2021). These lures were only developed for this species relatively recently, being released commercially in 2020. They provide a quick and efficient method for recording the species and have revolutionised understanding of its distribution, as have other lures developed for other clearwing species, e.g. ( Burman *et al.*, 2016). Many clearwing species are rarely observed as adults without lures, due to their similarity to the Hymenoptera they mimic, and the fact that they fly by day, but many (like *S. bembecifo*rmis) have reduced mouth-parts and do not visit flowers.

The main method for recording many species before the advent of lures has therefore been to search for the well-hidden larvae, and dedicated surveys for *S. bembeciformis* larval exit holes have revealed it from areas where it was previously entirely unknown ( Crégu, 2015; Goossens, 2020; Lastuvka & Lastuvka, 2014), and in some cases showed it to be very common. In the UK, it is the most widespread clearwing species, known from all areas ( Waring *et al.,* 2017). Outside the UK it has a limited, mostly western European distribution ( Lastuvka & Lastuvka, 2001).

The genome of *Sesia bembeciformis* was sequenced as part of the Darwin Tree of Life Project, a collaborative effort to sequence all named eukaryotic species in the Atlantic Archipelago of Britain and Ireland. Here we present a chromosomally complete genome sequence for *Sesia bembeciformis*, based on one male specimen from Wytham Woods, Oxfordshire, UK.

### Genome sequence report

**Figure 1.**
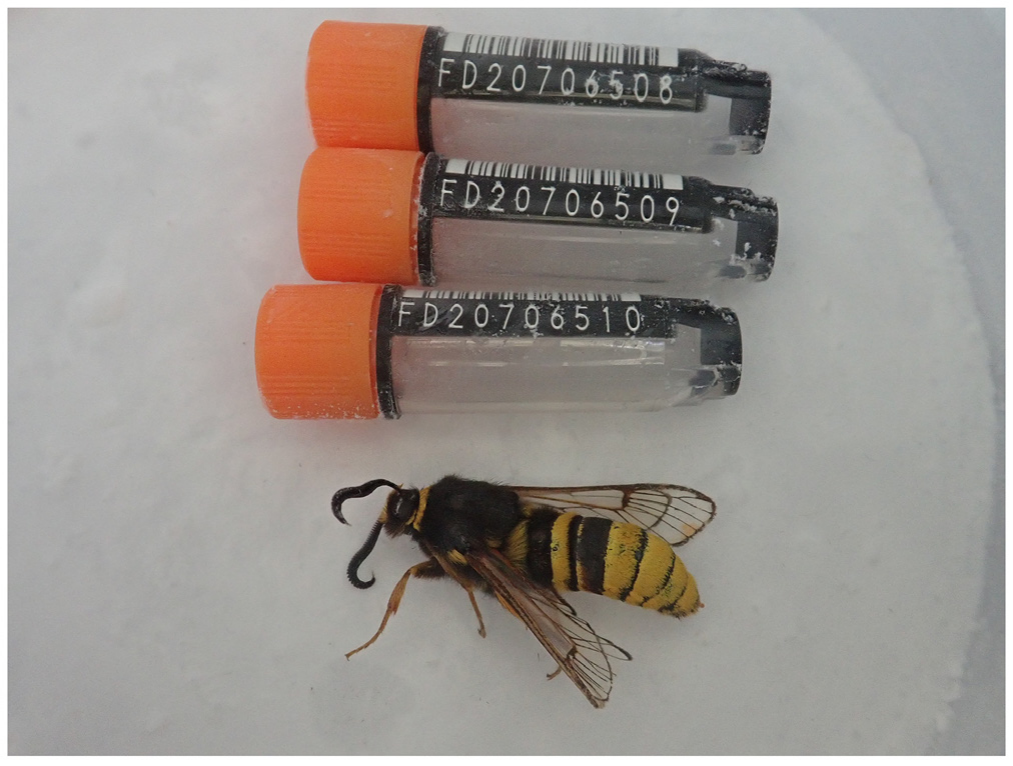
Photograph of the *Sesia bembeciformis* (ilSesBemb1) specimen used for genome sequencing.

The genome was sequenced from one male *S. bembeciformis* ( Figure 1) collected from Wytham Woods, Oxfordshire (latitude 51.775, longitude –1.315). A total of 27-fold coverage in Pacific Biosciences single-molecule HiFi long was generated. Primary assembly contigs were scaffolded with chromosome conformation Hi-C data. Manual assembly curation corrected four missing joins or mis-joins and removed ten haplotypic duplications, reducing the scaffold number by 18.52%.

**Figure 2.**
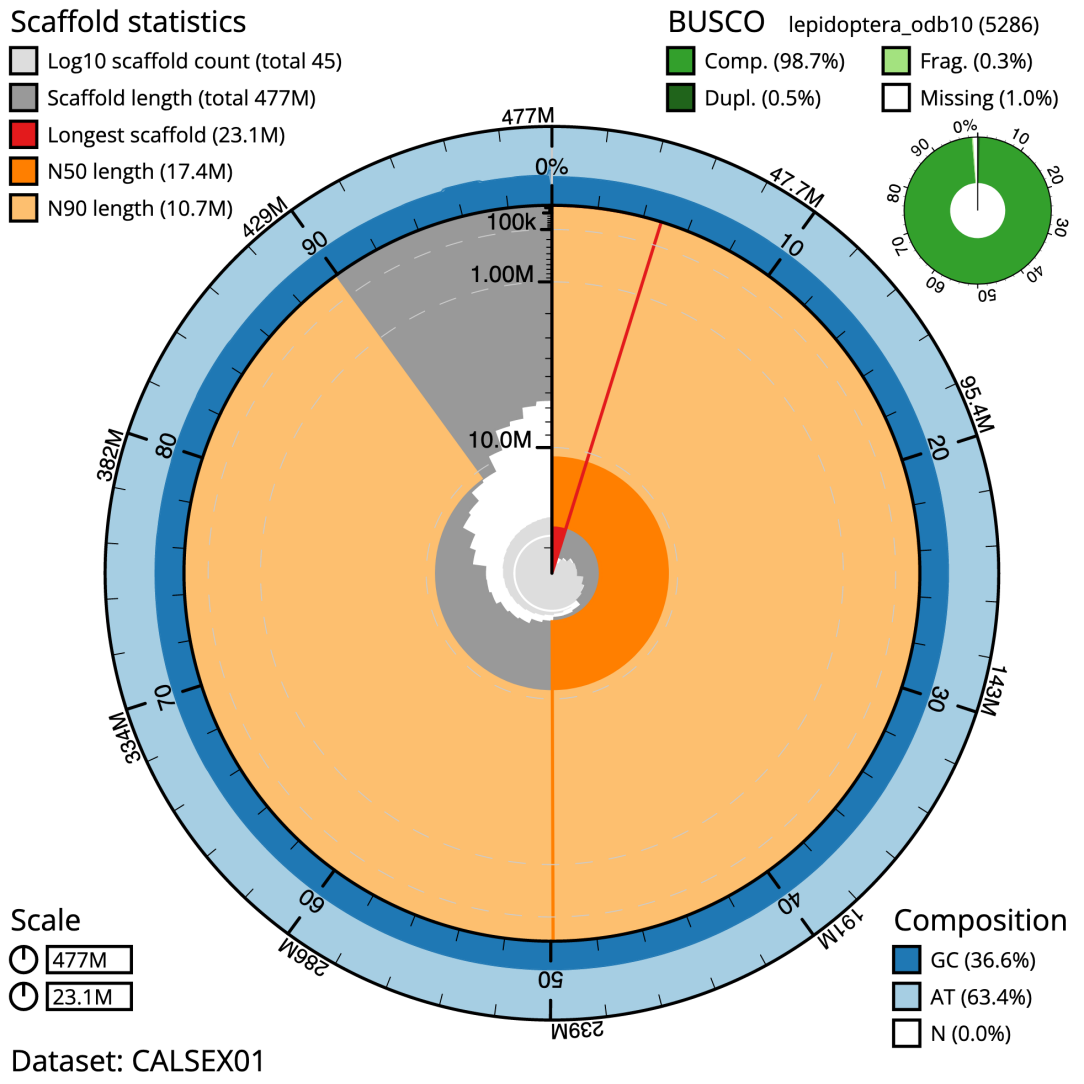
Genome assembly of *Sesia bembeciformis*, ilSesBemb1.1: metrics. The BlobToolKit Snailplot shows N50 metrics and BUSCO gene completeness. The main plot is divided into 1,000 size-ordered bins around the circumference with each bin representing 0.1% of the 477,151,446 bp assembly. The distribution of scaffold lengths is shown in dark grey with the plot radius scaled to the longest scaffold present in the assembly (23,076,235 bp, shown in red). Orange and pale-orange arcs show the N50 and N90 scaffold lengths (17,397,286 and 10,737,896 bp), respectively. The pale grey spiral shows the cumulative scaffold count on a log scale with white scale lines showing successive orders of magnitude. The blue and pale-blue area around the outside of the plot shows the distribution of GC, AT and N percentages in the same bins as the inner plot. A summary of complete, fragmented, duplicated and missing BUSCO genes in the lepidoptera_odb10 set is shown in the top right. An interactive version of this figure is available at https://blobtoolkit.genomehubs.org/view/ilSesBemb1.1/dataset/CALSEX01/snail.

The final assembly has a total length of 477.1 Mb in 44 sequence scaffolds with a scaffold N50 of 17.4 Mb ( Table 1). Most (99.99%) of the assembly sequence was assigned to 31 chromosomal-level scaffolds, representing 30 autosomes and the Z sex chromosome. Chromosome-scale scaffolds confirmed by the Hi-C data are named in order of size. ( Figure 2– Figure 5; Table 2). The assembly has a BUSCO v5.3.2 ( Manni *et al.*, 2021) completeness of 98.7% (single 98.2%, duplicated 0.5%) using the lepidoptera_odb10 reference set. While not fully phased, the assembly deposited is of one haplotype. Contigs corresponding to the second haplotype have also been deposited.

**Table 1.** Genome data for *Sesia bembeciformis*, ilSesBemb1.1.

Project accession data		
Assembly identifier	ilSesBemb1.1	
Species	*Sesia bembeciformis*	
Specimen	ilSesBemb1	
NCBI taxonomy ID	287197	
BioProject	PRJEB52659	
BioSample ID	SAMEA10978929	
Isolate information	male, ilSesBemb1 (PacBio and Hi-C) male, ilSesBemb2 (RNA-Seq)	
Assembly metrics ^*^		*Benchmark*
Consensus quality (QV)	64.7	*≥ 50*
*k*-mer completeness	100%	*≥ 95%*
BUSCO ^**^	C:98.7%[S:98.2%,D:0.5%], F:0.3%,M:1.0%,n:5,286	*C ≥ 95%*
Percentage of assembly mapped to chromosomes	99.99%	*≥ 95%*
Sex chromosomes	Z chromosome	*localised homologous pairs*
Organelles	Mitochondrial genome assembled	*complete single alleles*
Raw data accessions		
PacificBiosciences SEQUEL II	ERR9793194	
Hi-C Illumina	ERR9710923	
PolyA RNA-Seq Illumina	ERR10123699	
Genome assembly		
Assembly accession	GCA_943735995.1	
*Accession of alternate haplotype*	GCA_943735985.1	
Span (Mb)	477.1	
Number of contigs	51	
Contig N50 length (Mb)	17.1	
Number of scaffolds	44	
Scaffold N50 length (Mb)	17.4	
Longest scaffold (Mb)	23.1	
Genome annotation		
Number of protein-coding genes	15,843	
Number of gene transcripts	16,010	

* Assembly metric benchmarks are adapted from column VGP-2020 of “Table 1: Proposed standards and metrics for defining genome assembly quality” from ( Rhie *et al*., 2021). ** BUSCO scores based on the lepidoptera_odb10 BUSCO set using v5.3.2. C = complete [S = single copy, D = duplicated], F = fragmented, M = missing, n = number of orthologues in comparison. A full set of BUSCO scores is available at https://blobtoolkit.genomehubs.org/view/ilSesBemb1.1/dataset/CALSEX01/busco..

**Figure 3.**
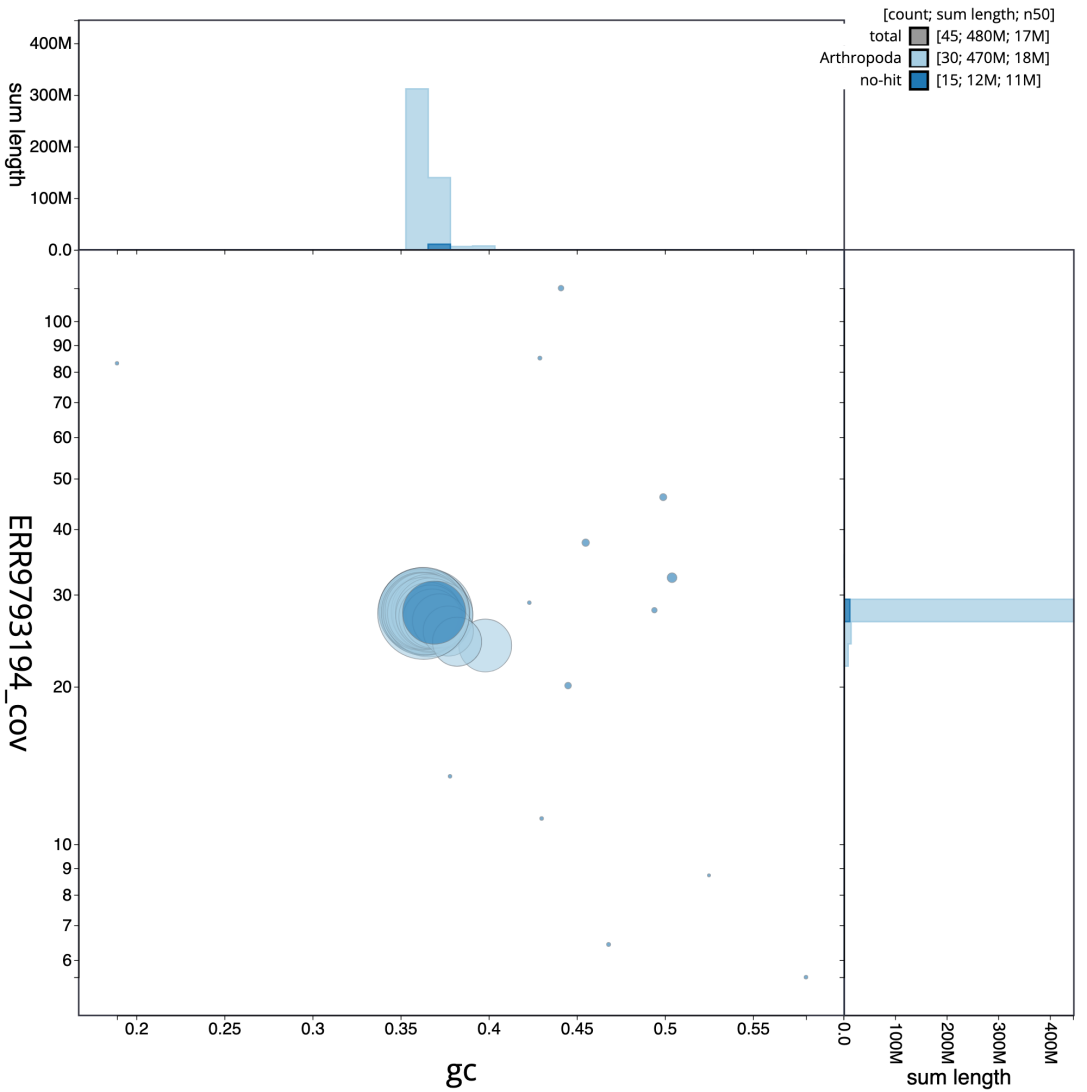
Genome assembly of *Sesia bembeciformis*, ilSesBemb1.1: GC coverage. BlobToolKit GC-coverage plot. Scaffolds are coloured by phylum. Circles are sized in proportion to scaffold length. Histograms show the distribution of scaffold length sum along each axis. An interactive version of this figure is available at https://blobtoolkit.genomehubs.org/view/ilSesBemb1.1/dataset/CALSEX01/blob.

**Figure 4.**
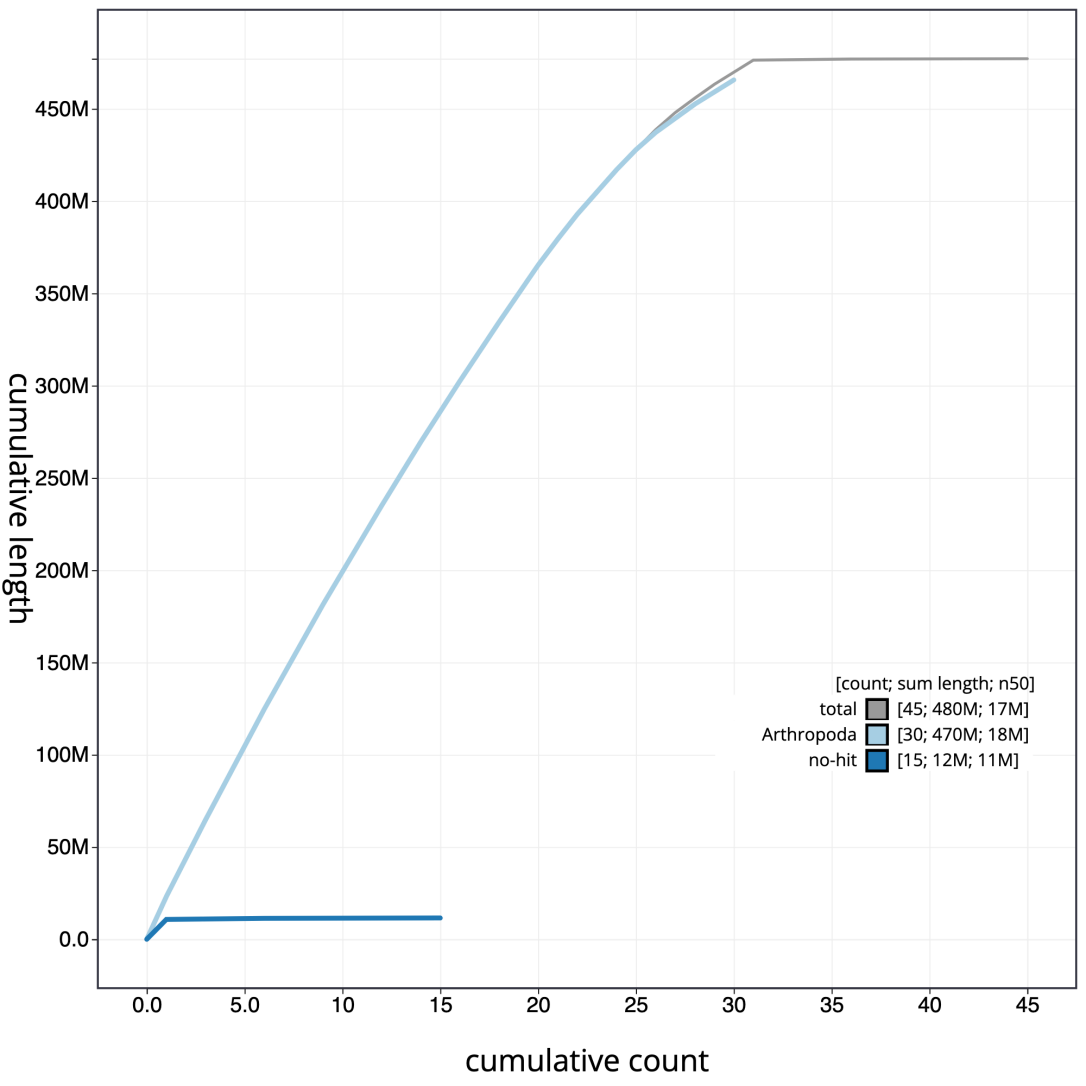
Genome assembly of *Sesia bembeciformis*, ilSesBemb1.1: cumulative sequence. BlobToolKit cumulative sequence plot. The grey line shows cumulative length for all scaffolds. Coloured lines show cumulative lengths of scaffolds assigned to each phylum using the buscogenes taxrule. An interactive version of this figure is available at https://blobtoolkit.genomehubs.org/view/ilSesBemb1.1/dataset/CALSEX01/cumulative.

**Figure 5.**
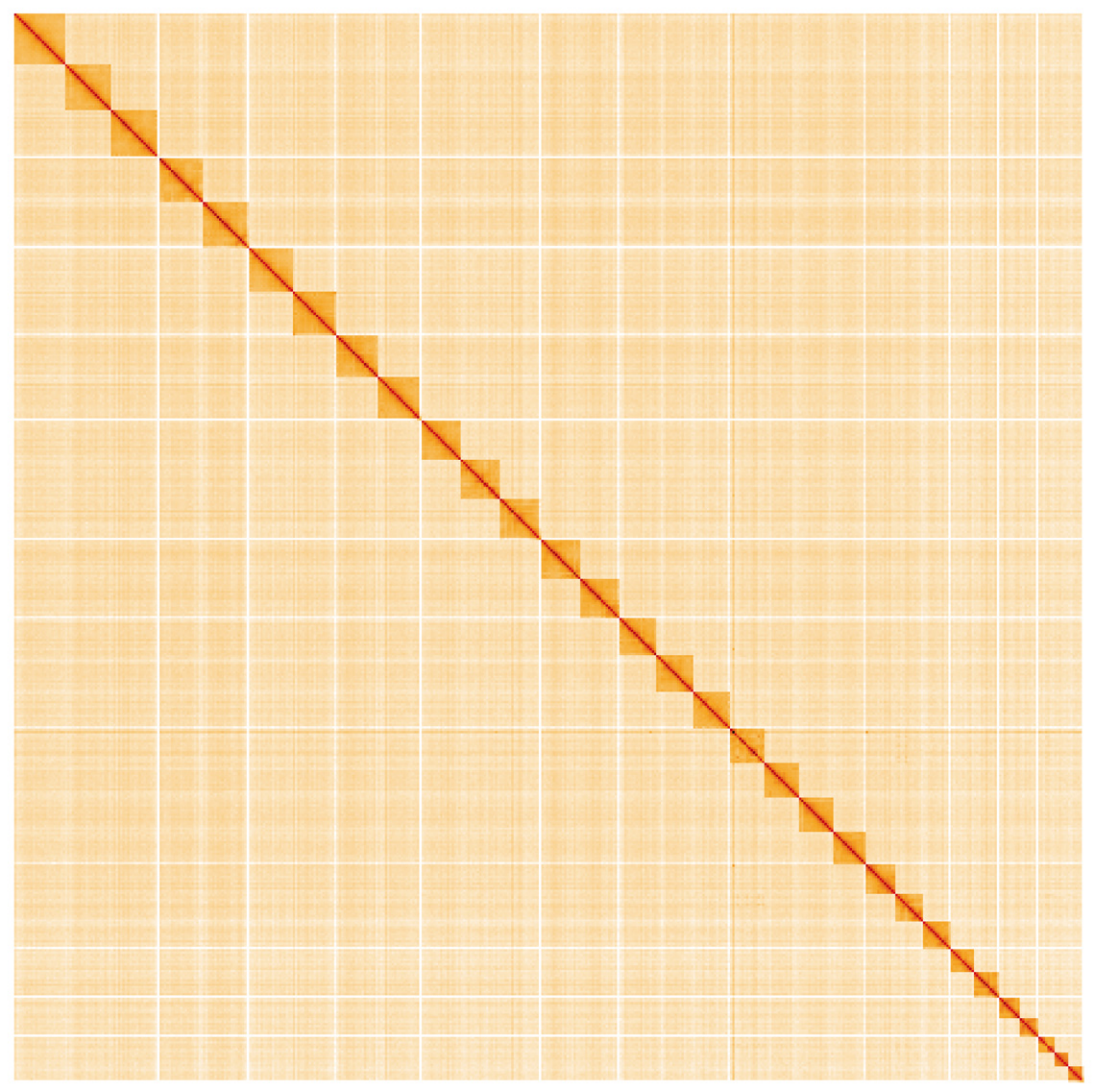
Genome assembly of *Sesia bembeciformis*, ilSesBemb1.1: Hi-C contact map. Hi-C contact map of the ilSesBemb1.1 assembly, visualised using HiGlass. Chromosomes are shown in order of size from left to right and top to bottom. An interactive version of this figure may be viewed at https://genome-note-higlass.tol.sanger.ac.uk/l/?d=UU22HbFPTsSHjXIUIZh37w.

### Genome annotation report

The *S. bembeciformis* GCA_943735995.1 genome assembly was annotated using the Ensembl rapid annotation pipeline ( Table 1; https://rapid.ensembl.org/Sesia_bembeciformis_GCA_943735995.1/). The resulting annotation includes 16,010 transcribed mRNAs from 15,843 protein-coding genes.

**Table 2.** Chromosomal pseudomolecules in the genome assembly of *Sesia bembeciformis*, ilSesBemb1.

INSDC accession	Chromosome	Size (Mb)	GC%
OX031025.1	1	20.93	36.6
OX031026.1	2	20.62	36.3
OX031027.1	3	20.06	36.5
OX031028.1	4	19.97	36.4
OX031029.1	5	19.91	36.2
OX031030.1	6	19.17	36.2
OX031031.1	7	18.84	36.1
OX031032.1	8	18.69	36.3
OX031033.1	9	17.98	36.5
OX031034.1	10	17.83	36.2
OX031035.1	11	17.81	36.2
OX031036.1	12	17.4	36.3
OX031037.1	13	17.14	36.4
OX031038.1	14	16.6	36.4
OX031039.1	15	16.42	36.2
OX031040.1	16	16.21	36.3
OX031041.1	17	15.71	36.7
OX031042.1	18	15.5	36.7
OX031043.1	19	15.44	36.6
OX031044.1	20	14.08	36.3
OX031045.1	21	13.46	36.7
OX031046.1	22	12.15	37.2
OX031047.1	23	11.89	36.6
OX031048.1	24	10.94	36.7
OX031049.1	25	10.74	36.9
OX031050.1	26	9.36	36.8
OX031051.1	27	7.49	39.8
OX031052.1	28	7.8	37.2
OX031053.1	29	6.74	37.7
OX031054.1	30	6.45	38.2
OX031024.1	Z	23.08	36.3
OX031055.1	MT	0.02	19.8
-	unplaced	0.75	47.5

## Methods

### Sample acquisition and nucleic acid extraction

Two male *Sesia bembeciformis* specimens (ilSesBemb1 and ilSesBemb2) were collected from Wytham Woods, Oxfordshire (biological vice-county Berkshire) (latitude 51.775, longitude –1.315) on 17 July 2021. The specimens were taken from an orchard by Douglas Boyes (University of Oxford) using a *pheromone* lure. The specimens were identified by Douglas Boyes and snap-frozen on dry ice.

DNA was extracted at the Tree of Life laboratory, Wellcome Sanger Institute (WSI). The ilSesBemb1 sample was weighed and dissected on dry ice with tissue set aside for Hi-C sequencing. Abdomen tissue was cryogenically disrupted to a fine powder using a Covaris cryoPREP Automated Dry Pulveriser, receiving multiple impacts. High molecular weight (HMW) DNA was extracted using the Qiagen MagAttract HMW DNA extraction kit. HMW DNA was sheared into an average fragment size of 12–20 kb in a Megaruptor 3 system with speed setting 30. Sheared DNA was purified by solid-phase reversible immobilisation using AMPure PB beads with a 1.8X ratio of beads to sample to remove the shorter fragments and concentrate the DNA sample. The concentration of the sheared and purified DNA was assessed using a Nanodrop spectrophotometer and Qubit Fluorometer and Qubit dsDNA High Sensitivity Assay kit. Fragment size distribution was evaluated by running the sample on the FemtoPulse system.

RNA was extracted from abdomen tissue of ilSesBemb2 in the Tree of Life Laboratory at the WSI using TRIzol, according to the manufacturer’s instructions. RNA was then eluted in 50 μl RNAse-free water and its concentration assessed using a Nanodrop spectrophotometer and Qubit Fluorometer using the Qubit RNA Broad-Range (BR) Assay kit. Analysis of the integrity of the RNA was done using Agilent RNA 6000 Pico Kit and Eukaryotic Total RNA assay.

### Sequencing

Pacific Biosciences HiFi circular consensus and 10X Genomics read cloud DNA sequencing libraries were constructed according to the manufacturers’ instructions. Poly(A) RNA-Seq libraries were constructed using the NEB Ultra II RNA Library Prep kit. DNA and RNA sequencing were performed by the Scientific Operations core at the WSI on Pacific Biosciences SEQUEL II (HiFi) and Illumina NovaSeq 6000 (RNA-Seq). Hi-C data were also generated from abdomen tissue of ilSesBemb1 using the Arima v2 kit and sequenced on the Illumina NovaSeq 6000 instrument.

### Genome assembly

Assembly was carried out with Hifiasm ( Cheng *et al.*, 2021). The assembly was scaffolded with Hi-C data ( Rao *et al.*, 2014) using YaHS ( Zhou *et al.*, 2023). The assembly was checked for contamination as described previously ( Howe *et al.*, 2021). Manual curation was performed using HiGlass ( Kerpedjiev *et al.*, 2018) and Pretext ( Harry, 2022). The mitochondrial genome was assembled using MitoHiFi ( Uliano-Silva *et al.*, 2022), which performed annotation using MitoFinder ( Allio *et al.*, 2020). The genome was analysed and BUSCO scores were generated within the BlobToolKit environment ( Challis *et al.*, 2020). Table 3 contains a list of all software tool versions used, where appropriate.

**Table 3.** Software tools and versions used.

Software tool	Version	Source
BlobToolKit	3.5.2	Challis *et al.,* 2020
Hifiasm	0.16.1-r375	Cheng *et al.,* 2021
HiGlass	1.11.6	Kerpedjiev *et al.,* 2018
MitoHiFi	2	Uliano-Silva *et al.,* 2022
PretextView	0.2	Harry, 2022
YaHS	yahs-1.1.91eebc2	Zhou *et al.,* 2023

### Genome annotation

The BRAKER2 pipeline ( Brůna *et al.*, 2021) was used in the default protein mode to generate annotation for the *S. bembeciformis* assembly (GCA_943735995.1) in Ensembl Rapid Release.

### Ethics and compliance issues

The materials that have contributed to this genome note have been supplied by a Darwin Tree of Life Partner. The submission of materials by a Darwin Tree of Life Partner is subject to the Darwin Tree of Life Project Sampling Code of Practice. By agreeing with and signing up to the Sampling Code of Practice, the Darwin Tree of Life Partner agrees they will meet the legal and ethical requirements and standards set out within this document in respect of all samples acquired for, and supplied to, the Darwin Tree of Life Project. All efforts are undertaken to minimise the suffering of animals used for sequencing. Each transfer of samples is further undertaken according to a Research Collaboration Agreement or Material Transfer Agreement entered into by the Darwin Tree of Life Partner, Genome Research Limited (operating as the Wellcome Sanger Institute), and in some circumstances other Darwin Tree of Life collaborators.

## Data Availability

European Nucleotide Archive: *Sesia bembeciformis* (lunar hornet). Accession number PRJEB52659; https://identifiers.org/ena.embl/PRJEB52659. ( Wellcome Sanger Institute, 2022) The genome sequence is released openly for reuse. The *Sesia bembeciformis* genome sequencing initiative is part of the Darwin Tree of Life (DToL) project. All raw sequence data and the assembly have been deposited in INSDC databases. Raw data and assembly accession identifiers are reported in Table 1.

## Author information

Members of the University of Oxford and Wytham Woods Genome Acquisition Lab are listed here: https://doi.org/10.5281/zenodo.4789928.

Members of the Darwin Tree of Life Barcoding collective are listed here: https://doi.org/10.5281/zenodo.4893703.

Members of the Wellcome Sanger Institute Tree of Life programme are listed here: https://doi.org/10.5281/zenodo.4783585.

Members of Wellcome Sanger Institute Scientific Operations: DNA Pipelines collective are listed here: https://doi.org/10.5281/zenodo.4790455.

Members of the Tree of Life Core Informatics collective are listed here: https://doi.org/10.5281/zenodo.5013541.

Members of the Darwin Tree of Life Consortium are listed here: https://doi.org/10.5281/zenodo.4783558.
