# Supplementary material for: The genome sequence of the Lunar Hornet, Sesia bembeciformis (Hübner 1806)
Source: Wellcome Open Res. 2023 Mar 1;8:109. [Version 1] doi: 10.12688/wellcomeopenres.19111.1 (PMC10568212; doi:10.12688/wellcomeopenres.19111.1)
Supplement: Peer review history

# Reviewer report: Reviewer response for version 1

**DOI:** 10.21956/wellcomeopenres.21187.r67882

**Published:** 2023-10-11

**Authors:** Roman Wąsala

**Affiliations:** 1 Department of Entomology and Environment Protection, University of Life Sciences in Poznań, Dąbrowskiego, Poland

The authors present the genome sequence of the Lunar Hornet, *Sesia bembeciformis* (Hübner 1806) Two male *Sesia bembeciformi*s were obtained from Wytham Woods, Oxfordshire, on 17 July 2021. The specimens were collected from the orchard using a pheromone lure. This is the next Sesiidae genome to be sequenced. The *Sesia bembeciformis* genome was sequenced as part of the Darwin Tree of Life project. The chromosome scale is of the same high quality as other genomes generated by DtoL. The pipeline is identical to other genomes, which is great. The resulting annotation includes 16,010 transcribed mRNAs from 15,843 protein-coding genes. The introductory text is presented in a comprehensive and enjoyable manner for the reader. The photographs of the Sesia bembeciformis specimens could have been better or properly corrected in a suitable graphics programme.

Are sufficient details of methods and materials provided to allow replication by others?

Yes

Is the rationale for creating the dataset(s) clearly described?

Yes

Are the datasets clearly presented in a useable and accessible format?

Yes

Are the protocols appropriate and is the work technically sound?

Yes

Reviewer Expertise:

Broad Lepidopterology with particular emphasis on moths of the superfamily Noctuoidea

I confirm that I have read this submission and believe that I have an appropriate level of expertise to confirm that it is of an acceptable scientific standard.

# Reviewer report: Reviewer response for version 1

**DOI:** 10.21956/wellcomeopenres.21187.r67884

**Published:** 2023-10-05

**Authors:** Fahad Alqahtani

**Affiliations:** 1 King Abdulaziz City for Science and Technology, Riyadh, Saudi Arabia

The authors of "The genome sequence of the Lunar Hornet, Sesia bembeciformis (Hübner 1806)," have successfully reconstructed the genome sequence of the Lunar Hornet. The assembly quality was evaluated using BUSCO, leading to high scores. The genome assembly contains 31 chromosomal-level scaffolds, including 30 autosomes and the Z sex chromosome, totaling 477,100,000 base pairs. The background is well-organized, with a clear and logical flow throughout the content, showcasing a significant contribution to the field of genomics.

 However, there are a few minor issues that need to be addressed. 

- In the Methods section (Sequencing), the authors mentioned 10X Genomics read cloud DNA sequencing. However, the paper specified the use of Pacific Biosciences SEQUEL II, Hi-C Illumina, and PolyA RNA-Seq Illumina. Can you explain this ambiguity? 
- In the "Genome Sequence Report," the authors also stated that the genome was reconstructed from one male. However, in the Methods - Sample Acquisition and Nucleic Acid Extraction, it was mentioned that two males were collected. Can you explain this? 
- A third issue arises from the RNA-seq data, which was not mentioned in the genome assembly section. The genome assembly section requires more details, including the data used for Hifiasm (Pacbio data is known to be used), as I understand the manuscript should be reproducible. 
- Moreover, in the Methods section (Genome Assembly, Curation, and Evaluation), it would be valuable to specify the closely related species used when MitoHiFi/Mitofinder guided the annotation of the mitochondrial genome.
- Lastly, in the Methods section (Genome Assembly, Curation, and Evaluation), the term "Pretext" should align with the software tools table, which is mentioned as "PretextView."

Are sufficient details of methods and materials provided to allow replication by others?

Yes

Is the rationale for creating the dataset(s) clearly described?

Yes

Are the datasets clearly presented in a useable and accessible format?

Yes

Are the protocols appropriate and is the work technically sound?

Yes

Reviewer Expertise:

Bioinformatics

I confirm that I have read this submission and believe that I have an appropriate level of expertise to confirm that it is of an acceptable scientific standard, however I have significant reservations, as outlined above.

# Reviewer report: Reviewer response for version 1

**DOI:** 10.21956/wellcomeopenres.21187.r64898

**Published:** 2023-09-07

**Authors:** Benoit Nabholz

**Affiliations:** 1 Universite de Montpellier, Montpellier, Occitanie, France

The authors present the chromosome scale assembly of the Lunar Hornet, *Sesia bembeciformis*. It is the 16th Sesiidae genome to be sequenced (most of them by the Darwin Tree of Life project, DtoL).

 The genome is of the same high quality as the other genomes produced by the DtoL project. The pipeline is the same as for the other genomes which is excellent. All the links and datasets presented are publicly available.

 The text of the introduction reads well and is interesting.

 The photos of the specimens could have been better, i.e. more light, a scale and a side and dorsal photo.

Are sufficient details of methods and materials provided to allow replication by others?

Yes

Is the rationale for creating the dataset(s) clearly described?

Yes

Are the datasets clearly presented in a useable and accessible format?

Yes

Are the protocols appropriate and is the work technically sound?

Yes

Reviewer Expertise:

Molecular evolution

I confirm that I have read this submission and believe that I have an appropriate level of expertise to confirm that it is of an acceptable scientific standard.
